# Supplementary material for: Readiness of healthcare facilities with tuberculosis services to manage diabetes mellitus in Tanzania: A nationwide analysis for evidence-informed policy-making in high burden settings
Source: PLoS One. 2021 Jul 12;16(7):e0254349. doi: 10.1371/journal.pone.0254349 (PMC8274870; doi:10.1371/journal.pone.0254349)
Supplement: Attachment — Submitted filename: Reviewers comments.doc [file pone.0254349.s001.doc]

**General comments**

The study is well written

**Specific comments**

**Introduction part**

Line number 77, there has been an increased burden of TB-DM comorbidity the reference cited here reference 19 does not indicate that there is an increase in the burden of TB and DM, also reference number 20. May be the author is referring other studies, the author should cite the correct citation for this statement

Line 99-104

Sputum smear microscopy remains the gold standard for-----------------].

All these have nothing to do with the readiness of the TB facilities to provide DM care? So this is irrelevant in this introduction

Line 114 and 115. As the burden of TB/DM comorbidity is on rising in Tanzania there is a dearth of information about the capacity of tuberculosis facilities to manage diabetes.

Provide reference for this information

Materials and Methods

Study design is missing

Line 132, NCDs, where the survey able to establish weather or not the health facilities is read to deliver services for all NCDs or there were few selected NCDs like the selected communicable disease?

Line 168-170 The domains were; staff training and guideline, diagnostics (sputum smear microscopy), equipment (blood pressure machines, stethoscopes, weighing scale, and Stadiometer),

None of these mentioned here are used to diagnose DM. How DM was diagnosed. If the mentioned survey uses this tool to conclude that they diagnose DM it is wrong

Results

Table 1. When categorizing the Health facilities there is only public and Private, where are the FBO facilities? These are neither public nor private you need to categorize this

Line 233 when referring Clinic does this mean stand alone clinic or TB clinic located in a Dispensary, health center or Hospital? Please define this

When it comes to TB facilities there are two types according to Tanzania National TB program. There are TB facilities which offer both TB diagnosis and treatment (DOT) Services and other TB clinic which offer only treatment and this are referred to as DOTS center Indicate which one are referred here

Discussion section

Line 270-272 Based on facility characteristics, the majority of TB facilities with the availability of DM services were health centres and dispensaries, publicly owned, and rural located facilities

The author need to revisit the analysis section in Tanzania all hospitals have the basic facilities for diagnosis of DM, when saying majority of the facilities with DM diagnosis are HC and dispensary is because HC and DM were more than Hospitals in this analysis.

Page 15 last paragraph starting with line 288, the author need to explore more why the TB clinic are not ready to provide DM services/ In most cases TB facilities are under staffed just as the whole Health sector in Tanzania is under staffed, adding more burden of managing DM the staff will not be comfortable?
