# Supplementary material for: Readiness of healthcare facilities with tuberculosis services to manage diabetes mellitus in Tanzania: A nationwide analysis for evidence-informed policy-making in high burden settings
Source: PLoS One. 2021 Jul 12;16(7):e0254349. doi: 10.1371/journal.pone.0254349 (PMC8274870; doi:10.1371/journal.pone.0254349)
Supplement: Attachment — Submitted filename: Comments.docx [file pone.0254349.s002.docx]

Study title: **Readiness of Tuberculosis healthcare facilities to manage Diabetes mellitus in Tanzania: a nationwide analysis for evidence-informed policy-making in high burden settings**

The study addressed a very important aspect currently facing developing World; coexistence of a longtime infectious disease like Tuberculosis (TB) together with an emerging challenge of Non-Communicable disease like Diabetes (DM).

**Abstract:**

(i)**Introduction**: Include at least a line on the aim/objective(s) of the study (ii)**Conclusion:** First line of the conclusion: from your result section, none of the health facilities was ready for managing TB-DM coexistence. Hence the readiness was not low, but it was not there at al.

**Main body:**

(i)The introduction is too long for a typical scientific manuscript. Please consider including the following: Burden of TB, burden of DM, interaction of TB and DM, what is currently done/known in Tanzania regarding TB-DM management, what are the gaps intended to be filled by the proposed study and what is proposed to be done in the study.

(ii) Results: Line 223: I think you should clearly say something like “Majority of the health facilities were dispensaries and clinics (xx%)………………………………………

(iii) Discussion: The authors did a good job to discuss the implications of the findings. However, the authors need to review the discussion section to makes sure that there are repetitions of what has been reported in the result section. The discussion needs to tell the readers the interpretations of the findings.

Moreover, the authors did not tell the readers what other articles found about the same issues addressed in the article under discussion. The authors have the obligation to compare and contrast with other articles and discuss whatever they find in other articles.

(iv) Conclusion: The conclusion almost repeated result section. Please revise the conclusion to clearly state what is concluded as a result of the findings and the discussion of this article.
