# Supplementary material for: Readiness of healthcare facilities with tuberculosis services to manage diabetes mellitus in Tanzania: A nationwide analysis for evidence-informed policy-making in high burden settings
Source: PLoS One. 2021 Jul 12;16(7):e0254349. doi: 10.1371/journal.pone.0254349 (PMC8274870; doi:10.1371/journal.pone.0254349)
Supplement: Attachment — Submitted filename: Response to Reviewer`s comments.docx [file pone.0254349.s003.docx]

**Responses to Reviewers` comments**

**Reviewer 1:**

**General comment:**
The study is well written

**Response**

Thank you very much for taking the time to review our manuscripts. We appreciate your comments, recommendations and questions which have helped us to improve our manuscript as well for future write-up.

**Specific comments**

**Comment 1**

Line number 77, there has been an increased burden of TB-DM comorbidity the reference cited here reference 19 does not indicate that there is an increase in the burden of TB and DM, also reference number 20. May be the author is referring other studies, the author should cite the correct citation for this statement

**Response**

In response to the reviewer`s comments, the references have been revised and the respective section paraphrased to reflect the cited articles (Page 4 line 82). The background section has been revised hence some cited references sequence were changed.

**Comment 2**

Line 99-104

Sputum smear microscopy remains the gold standard for-----------------].

All these have nothing to do with the readiness of the TB facilities to provide DM care? So this is irrelevant in this introduction

**Response**

According to the reviewer`s suggestion, the section has been deleted.

**Comment 3**

Line 114 and 115. As the burden of TB/DM comorbidity is on rising in Tanzania there is a dearth of information about the capacity of tuberculosis facilities to manage diabetes.

Provide reference for this information

**Response**

According to the reviewer`s comment, the reference has been inserted (Page 4 line 82).

**Comment 4**

Study design is missing

**Response**

In response to the reviewer`s comment, the information about study design has been added (Page5 line 96).

**Comment 5**

Line 132, NCDs, where the survey able to establish weather or not the health facilities is read to deliver services for all NCDs or there were few selected NCDs like the selected communicable disease?

**Response**

According to the reviewer`s questions, not all NCDs were selected. The survey selected only the major four NCDs: Chronic Respiratory Diseases, Cardiovascular Diseases, Hypertension, and Diabetes Mellitus. The analyses of these NCDs have already published. Kindly see some of the articles below:

1. Are Tanzanian health facilities ready to provide management of chronic respiratory diseases? An analysis of national survey for policy implications. PloS one. 2019 Jan 7;14(1):e0210350.
2. Disparities in availability of services and prediction of the readiness of primary healthcare to manage diabetes in Tanzania. Primary care diabetes. 2021 Apr 1;15(2):365-71.
3. Preparedness of lower-level health facilities and the associated factors for the outpatient primary care of hypertension: Evidence from Tanzanian national survey. PloS one. 2018 Feb 15;13(2):e0192942.

**Comment 6**

Line 168-170 The domains were; staff training and guideline, diagnostics (sputum smear microscopy), equipment (blood pressure machines, stethoscopes, weighing scale, and Stadiometer),

None of these mentioned here are used to diagnose DM. How DM was diagnosed. If the mentioned survey uses this tool to conclude that they diagnose DM it is wrong

**Response**

In response to the reviewer`s comment, the section has been revised to include the diagnostic tools for Diabetes Mellitus assessed in that survey (Page 7 lines 141-142).

Regarding the equipment (blood pressure machines, stethoscopes, weighing scale, and Stadiometer) are used for disease monitoring and detection of complications. For instance, a blood pressure machine and stethoscope is for detecting hypertension and cardiorespiratory complications, respectively. The weighing scale and Stadiometer are for assessing body weight and body mass index in respect to cardio-metabolic parameters such as LDL cholesterol.

**Comment 7**

Table 1. When categorizing the Health facilities there is only public and Private, where are the FBO facilities? These are neither public nor private you need to categorize this

**Response**

According to the reviewer`s questions, the intention was to compare the public facilities vs. non-public facilities. Therefore, to make it clear for the readers, the term private has been replaced with "non-public" to include all other non-public facilities (Page 10 line 208). Also, it has been defined on Page 6 line 112; Page 10 lines 19-194).

**Comment 8**

Line 233 when referring Clinic does this mean stand alone clinic or TB clinic located in a Dispensary, health center or Hospital? Please define this

**Response**

According to the reviewer`s question, in this study, a clinic refers to a non-publicly owned health facility that provides outpatient curative services. Usually, it is a standalone health facility different from diseases specific clinics within hospitals. The operational definition has been added on Page 7 lines 146-148.

**Comment 9**

When it comes to TB facilities there are two types according to Tanzania National TB program. There are TB facilities which offer both TB diagnosis and treatment (DOT) Services and other TB clinic which offer only treatment and this are referred to as DOTS centre Indicate which one are referred here

**Response**

In response to the reviewer`s comments and question, the TB facilities referred in this study were those providing both diagnosis and treatment (Page 7 line 134-135).

**Comment 10**

Line 270-272 Based on facility characteristics, the majority of TB facilities with the availability of DM services were health centres and dispensaries, publicly owned, and rural located facilities. The author need to revisit the analysis section in Tanzania all hospitals have the basic facilities for diagnosis of DM, when saying majority of the facilities with DM diagnosis are HC and dispensary is because HC and DM were more than Hospitals in this analysis.

**Response**

In response to the reviewer`s comments and suggestions, the section has been revised and correction made (Page 13 lines 251-252, and page 14, lines 253-254).

**Comment 11**

Page 15 last paragraph starting with line 288, the author need to explore more why the TB clinic are not ready to provide DM services/ In most cases TB facilities are under staffed just as the whole Health sector in Tanzania is under staffed, adding more burden of managing DM the staff will not be comfortable?

**Response**

In response to the reviewer`s comments and question, the explanation has been provided on Page 14 lines 273-274 and page 15 lines 275-276, 284-290; and page 16 lines 296-297.

**Reviewer 2:**

**General comment:**
The study addressed a very important aspect currently facing developing World; coexistence of a longtime infectious disease like Tuberculosis (TB) together with an emerging challenge of Non-Communicable disease like Diabetes (DM).

**Response**

Thank you very much for taking the time to review our manuscripts. We appreciate your comments, recommendations and questions which have helped us to improve our manuscript as well for future write-up.

**Specific comments**

**Comment 1**

Include at least a line on the aim/objective(s) of the study

**Response**

In response to the reviewer`s suggestion, the aim/objective has been included (Page 1 lines 19-20).

**Comment 2**

First line of the conclusion: from your result section, none of the health facilities was ready for managing TB-DM coexistence. Hence the readiness was not low, but it was not there at al.

**Response**

According to the reviewer`s comments, the section has been revised for clarity (Page 2, lines 34-35 and 37-38). The conclusion is based on overall readiness while the sentence on the results included specific domain readiness.

**Comment 3**

The introduction is too long for a typical scientific manuscript. Please consider including the following: Burden of TB, burden of DM, interaction of TB and DM, what is currently done/known in Tanzania regarding TB-DM management, what are the gaps intended to be filled by the proposed study and what is proposed to be done in the study.

**Response**

In response to the reviewer`s comment and suggestions, the introduction has been revised and shortened (Page 3-5, lines 44-91).

**Comment 4**

Results: Line 223: I think you should clearly say something like “Majority of the health facilities were dispensaries and clinics (xx%)………………………………………

**Response**

In response to the reviewer`s suggestion, the sentence has been revised (Page 11, lines 213-215).

**Comment 5**

Discussion: The authors did a good job to discuss the implications of the findings. However, the authors need to review the discussion section to makes sure that there are repetitions of what has been reported in the result section. The discussion needs to tell the readers the interpretations of the findings.

Moreover, the authors did not tell the readers what other articles found about the same issues addressed in the article under discussion. The authors have the obligation to compare and contrast with other articles and discuss whatever they find in other articles.

**Response**

According to the reviewer`s comments and suggestions, there is only one related study found to compared and contrast with the current study (Page 14 and 15, lines 274-276). Moreover, the discussion has been revised to omit repetition of the results (Page 13, 14, 15 and 16; lines 251-261 and 267-297.

**Comment 6**

Conclusion: The conclusion almost repeated result section. Please revise the conclusion to clearly state what is concluded as a result of the findings and the discussion of this article.

**Response**

In response to the reviewer`s comments and suggestions, the conclusion has been revised (Page 16, lines 307-311).
