# Supplementary material for: Readiness of healthcare facilities with tuberculosis services to manage diabetes mellitus in Tanzania: A nationwide analysis for evidence-informed policy-making in high burden settings
Source: PLoS One. 2021 Jul 12;16(7):e0254349. doi: 10.1371/journal.pone.0254349 (PMC8274870; doi:10.1371/journal.pone.0254349)
Supplement: Attachment — Submitted filename: Comments_HIVD.docx [file pone.0254349.s004.docx]

Title: Readiness of Tuberculosis healthcare facilities to manage Diabetes mellitus in Tanzania: a nationwide analysis for evidence-informed policy-making in high burden settings

**Abstract**: Consider using “health facilities with TB treatment service rather than TB facilities (It should also be clear if TB facilities referred to health facilities with TB treatment or TB diagnosis?)
